# Epigenetic clock in the aorta and age-related endothelial dysfunction in mice

**DOI:** 10.1007/s11357-024-01086-3

**Published:** 2024-02-21

**Authors:** Ewelina Pośpiech, Anna Bar, Aleksandra Pisarek-Pacek, Agnieszka Karaś, Wojciech Branicki, Stefan Chlopicki

**Affiliations:** 1 Department of Forensic Genetics, Pomeranian Medical University in Szczecin, Al. Powstancow Wielkopolskich 72, 70-204 Szczecin, Poland; 2 Jagiellonian Centre for Experimental Therapeutics (JCET), Jagiellonian University, Bobrzynskiego 14, 30-348 Krakow, Poland; 3 Institute of Zoology and Biomedical Research, Faculty of Biology, Jagiellonian University, Gronostajowa 9, 30-387 Krakow, Poland; 4 Malopolska Centre of Biotechnology, Jagiellonian University, Gronostajowa 7a, 30-387 Krakow, Poland; 5 Institute of Forensic Research, Westerplatte 9, 31-033 Kraków, Poland; 6 Faculty of Medicine, Chair of Pharmacology, Jagiellonian University Medical College, Grzegorzecka 16, 31-531 Krakow, Poland

**Keywords:** Epigenetic clock, Endothelial dysfunction, Ageing, Magnetic resonance imaging

## Abstract

While epigenetic age (EA) of mouse blood can be determined using DNA methylation analysis at three CpG sites in the *Prima1*, *Hsf4* and *Kcns1* genes it is not known whether this approach is useful for predicting vascular biological age. In this study we validated the 3-CpG estimator for age prediction in mouse blood, developed a new predictive model for EA in mouse aorta, and assessed whether epigenetic age acceleration (EAA) measured with blood and aorta samples correlates with age-dependent endothelial dysfunction. Endothelial function was characterized in vivo by MRI in 8–96-week-old C57BL/6 mice. Arterial stiffness was measured by USG-doppler. EA-related changes within 41 CpG sites in *Prima1*, *Kcns1* and *Hsf4* loci, were analyzed in the aorta and blood using bisulfite amplicon high-throughput sequencing. Progressive age-dependent endothelial dysfunction and changes in arterial stiffness were observed in 36-96-week-old C57BL/6 mice. Methylation levels of the investigated loci correlated with chronological age in blood and the aorta. The new model for EA estimation in aorta included three cytosines located in the *Kcns1* and *Hsf4*, explained R^2^ = 87.8% of the variation in age, and predicted age with an mean absolute error of 9.6 weeks in the independent test set. EAA in the aorta was associated with endothelial dysfunction in the abdominal aorta and femoral artery what was consistent with the EAA direction estimated in blood samples. The rate of vascular biological ageing in mice, reflected by the age-dependent systemic endothelial dysfunction, could be estimated using DNA methylation measurements at three loci in aorta and blood samples.

## Introduction

Ageing is the single most important risk factor for cardiovascular disease (CVD), such as hypertension, atherosclerosis and heart failure [1, 2]. In fact, the risk of CVD doubles every 10 years after the age of 40, even after adjusting for other risk factors (e.g., smoking, hypertension etc.) [3]. Therefore, targeting the biological mechanisms of ageing, particularly accelerated biological ageing, appears to be an important strategy for treating or preventing chronic, age-related diseases like CVD [3–5]. In particular, endothelial dysfunction of the large arteries represents the age-associated arterial phenotypes that predict CVD-related morbidity and mortality [6–8].

However, there is increasing evidence that progressive changes in the methylome can be used to track biological ageing. Many epigenetic biomarkers of age have been developed for human samples, which vary in the number of CpG markers included, applicability to specific tissues, and the strength of association with lifespan and health [9]. The development of the first epigenetic clocks [10, 11], which involved the analysis of dozens to hundreds of markers, was quickly followed by the development of more compact tools, based on the analysis of a few critical markers showing very high correlation with age [12, 13]. Importantly, epigenetic age acceleration (EAA), which reflects the difference between epigenetic age and chronological age, has been proposed as a contributor to several age-related health conditions, such as obesity, all-cause mortality, physical and cognitive fitness, cardiovascular health, and other age-related diseases [14–16].

DNA methylation (DNAm) has been shown to correlate with age in mice, but the marker set of CpG predictors is usually different from that used in humans [17, 18]. Additionally, a universal mammalian clock relying on cytosines that change their pattern with age has also been documented in a number of species in previous studies [19]. However, such models involve hundreds of CpGs. Therefore, a model for predicting epigenetic age in mouse blood samples using only three cytosines has been developed, which was first trained using pyrosequencing data [20] and later using bisulfite amplicon NGS data with a slightly modified set of CpGs within the same loci [21]. The 3-CpG age estimator was found to predict the chronological age of blood from C57BL/6 mice with an accuracy of ~ 7 weeks. Interestingly, increased levels of EAA have been shown in DBA/2 mice, which are known to have a shorter lifespan [20].

Here, we validated the 3-CpG model using blood samples from C57BL/6 mice and developed a new biomarker for age based on aortic samples trained on methylation data measured using high-throughput DNA sequencing technology. Furthermore, we verified the association between EAA and age-dependent deterioration of endothelial function, which we assessed in vivo using magnetic-resonance imaging (MRI).

## Materials and methods

### Animals

This study used 8-, 24-, 36-, 48- and 96-week-old C57BL/6 male mice (from Janvier Labs, Rodent Research Models & Associated Services, Le Genest-Saint-Isle, France and the Mossakowski Medical Research Centre, Polish Academy of Sciences, Warsaw, Poland). The size of the experimental groups is reported in the legends of the corresponding graphs. Mice were housed in collective cages, in a room with constant environmental conditions (22–25 °C, 65–75% humidity and 12 h light/dark cycle). Animals had ad libitum access to daily provided chow diet and water. All experiments were approved by the Local Ethics Committee of Jagiellonian University (Krakow, Poland) and were in accordance with the Guide for the Care and Use of Laboratory Animals of the National Academy of Sciences (NIH publication No. 85–23, revised 1996), as well as the Guidelines for Animal Care and Treatment of the European Community.

### Assessment of endothelial function and aortic pulse wave velocity (PWV) in vivo

**Fig. 1:**
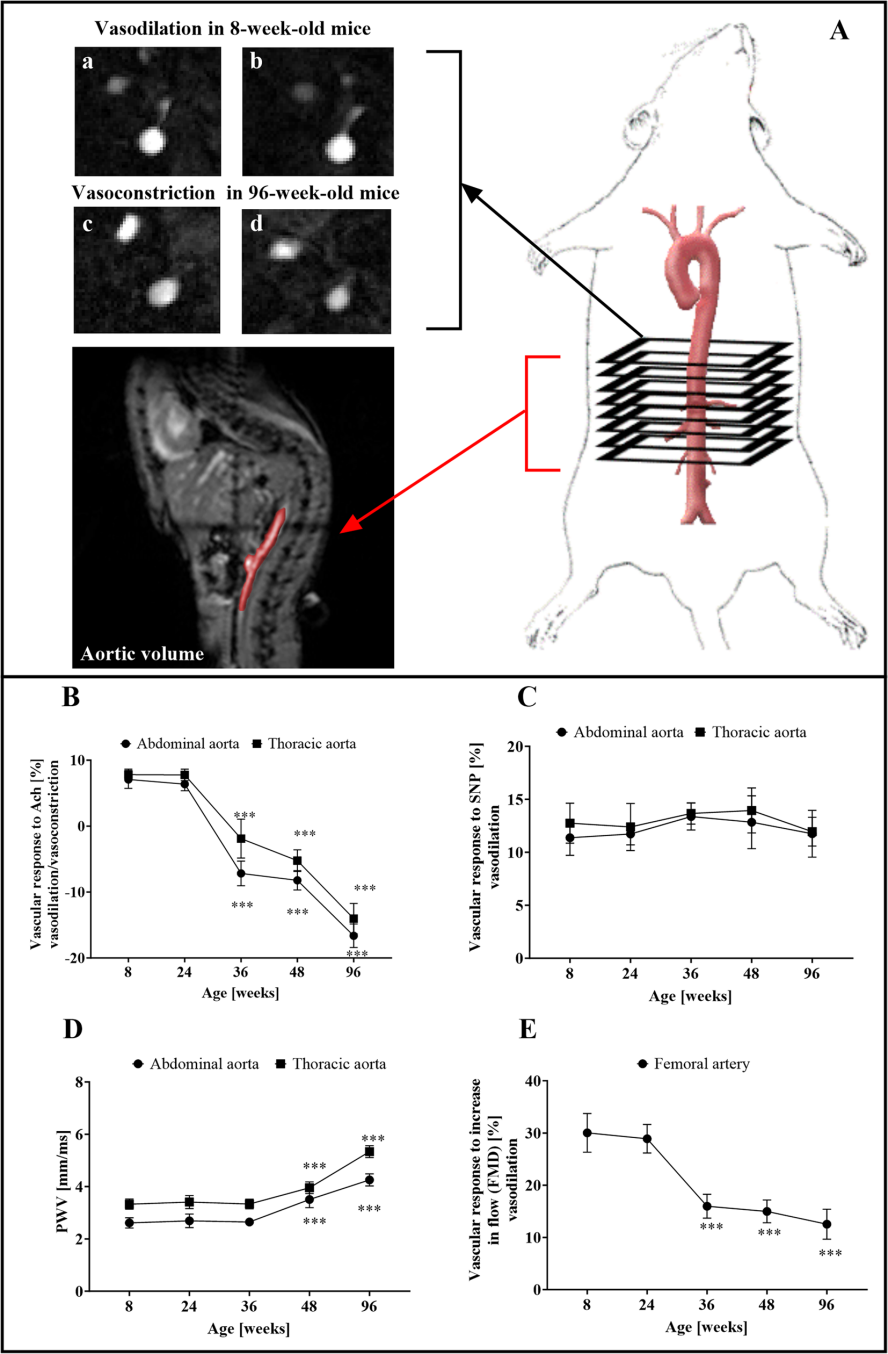
Age-dependent deterioration of endothelium-dependent vasodilation and development of arterial stiffness in C57BL/6 mice measured in vivo. (**A**) Scheme showing the principle of the magnetic resonance imaging (MRI)–based in vivo detection of endothelium-dependent vascular response induced by acetylcholine (Ach) with representative images depicting Ach-induced vasodilation in 8-week-old (a,b before and after Ach, respectively) as well as Ach-induced vasoconstriction in 96-week-old C57BL/6 mice (c,d before and after Ach, respectively). Changes in the vessel volume in response to Ach ( **B**) and to sodium nitroprusside (SNP, **C**), as well as pulse wave velocity (PWV, **D**), measured in the abdominal aorta (AA) and thoracic aorta (TA), and changes in the vessel volume in response to increase in flow in the femoral artery (FMD, **E**) in 8-, 24-, 36-, 48- and 96-week-old C57BL/6 control male mice (*n* = 6). Statistics: 1-way ANOVA with Post-Hoc Test Tukey's (normality was assessed using the Shapiro–Wilk test; ns – non-significant, *** = *p* < 0.001 in comparison to 8-week-old C57BL/6 mice).

Endothelial function was assessed by MRI in vivo, as described previously [22, 23]. During the MRI experiment, which was performed using a 9.4 T scanner (BioSpec 94/20 USR, Bruker, Germany), mice were imaged in the supine position under stable anesthesia (isoflurane (Aerrane, Baxter Sp. z o. o., Poland, 1.5 vol%) in oxygen and air (1:2) mixture). Heart function, respiration and body temperature (maintained at 37 °C using circulating warm water) were monitored using a Monitoring and Gating System (SA Inc., Stony Brook, NY, USA). Briefly, endothelium-dependent response to acetylcholine (Ach, Sigma-Aldrich, Poznań Poland: 50 μl, 16.6 mg/kg, i.p.) and endothelium-independent responses to sodium nitroprusside (SNP, Sigma-Aldrich, 1 mg/kg; i.v.) were assessed in the abdominal (AA) and thoracic (TA) areas of the aorta (Fig. 1A), while flow-mediated dilatation (FMD) in response to reactive hyperaemia was assessed in the femoral artery (FA). Imaging parameters, data acquisition and image analysis were conducted as described previously [22, 23].

PWV measurements were performed using a Doppler flow velocity system (DFVS, Indus Instruments, Scintica Instrumentation, Maastricht, The Netherlands) [24], with two 20-MHz Doppler probes, by simultaneously recording velocity signals from two sites that are representative of the TA and AA as described previously [23].

### Statistical analysis

The data are presented as mean and standard deviation, or in case of the lack of normal distribution as median and interquartile range. Statistical tests were performed using GraphPad Prism (GraphPad Software, Inc., USA). Non-parametric (Kruskal–Wallis test) and parametric (one-way analysis of variance (ANOVA) with honest significant difference (HSD) Tukey's test for unequal sample sizes) tests were performed. A value of *p* <  = 0.05 was considered to be statistically significant.

### Amplicon-based high-throughput sequencing protocol for DNA methylation data collection

After in vivo measurements, the mice were euthanized (100 mg/kg b.w. ketamine + 10 mg/kg b.w. xylazine, *i.p.*) and blood was drawn from the heart and collected in tubes containing 10% solution of ethylenediaminetetraacetic acid dipotassium salt (K_2_EDTA, Aqua-Med, Łodź, Poland, 1 μL of K_2_EDTA/100 μL of blood). After blood collection, the thoracic section of the aorta was isolated and cleared from the surrounding tissue. The obtained plasma and aorta samples were deep-frozen at – 80 °C. DNA was isolated from blood and aorta samples using the Sherlock AX Kit (A&A Biotechnology) and the QIAamp DNA Mini Kit (Qiagen), respectively. After qualitative and quantitative DNA evaluation, DNA was subjected to bisulfite conversion using the EZ DNA Methylation-Direct Kit (Zymo Research). Chemical modification with sodium bisulfite was then performed on 500 ng DNA, or less if the concentration of the DNA isolate was lower but met the minimum required amount of DNA of 200 ng. PCR amplification was performed separately for each target using 12.5 µl QIAGEN Multiplex PCR Master Mix (Qiagen), 2.5 µl primer mix, 7.5 µl PCR grade water, 2. 5 µl of converted DNA and the following thermocycler protocol: an initial denaturation step at 95˚C for 15 min, 38 cycles consisting of 94 ˚C for 30 s, 59 ˚C for 30 s, 72 ˚C for 1 min and a final extension step at 72 ˚C for 10 min. The sequences of the primers were derived from previous literature [21]. Then, 16.7 ng of PCR products for each of the three targets purified using 1.5X Agencourt AMPure XP beads were pooled and a total DNA input of 50 ng was subjected to library preparation in half of the reaction volume using the KAPA HyperPrep Kit and KAPA Unique-Dual Indexed (UDI) Adapters (Roche). Targeted sequencing was then conducted using the MiSeq Reagent Kit v3 (600 cycles) with 15 pM of library concentration and 5% PhiX added. Raw sequencing data in FASTQ files were quality controlled, trimmed and analysed as described in [13]. Reads mapped to the bisulfite-converted mm10.fasta reference were then used to measure DNA methylation levels, presented in % and calculated as the ratio of C reads to the sum of C and T reads.

### Age prediction modelling

The original coefficients for *Prima1* chr12:103,214,640, *Hsf4* chr8:105,271,022 and *Kcns1* chr2:164,168,063 reported by Han et al. [21] were used to determine epigenetic age in blood samples collected from C57BL/6 mice in the present study and to validate the performance of the model. The correlation between chronological age and DNA methylation levels at 41 investigated CpGs within three loci was analyzed in a training set of *N* = 24 aortic samples, including two biological replicates at each time point. Methylation data generated for a training set of aortic samples were used to develop a new age prediction model, with the selection of a set of optimal markers performed with a stepwise linear regression using a statistical test of model error improvement as the criterion for predictor entry or removal. The developed model was then assessed with an independent set of *N* = 12 aortic samples, including one biological replicate at each time point. Predictive accuracy was evaluated using the mean absolute error (MAE) parameter. For both models, Han et al. (blood) [21] and the new estimator (aorta) EAAs were determined and calculated as residuals from a linear regression analysis with epigenetic age defined as the dependent variable and chronological age as the independent variable. EAA was tested for association with characteristics of endothelial dysfunction, including the AA and TA diastole, femoral artery endothelial dysfunction, and the AA and TA stiffness, using linear regression with results corrected for chronological age. Analyses were performed with PS IMAGO PRO 9 software (IBM SPSS Statistics 29).

## Results

### Age-dependent impairment of endothelium-dependent vasodilation

In 8- and 24-week-old C57BL/6 mice, endothelial function was preserved as indicated by the magnitude of Ach-induced vasodilation in the AA and TA, which amounted to approximately 20%, and the flow-induced vasodilation in the FA, which amounted to approximately 30%. The progressive, age-dependent impairment of Ach-induced vasodilation was visible in 36-week-old and older mice (Fig. 1B) and featured by the loss of vasodilator response and paradoxical vasoconstriction observed in the AA (-7.17%, -8.22% and -16.64% for 36-, 48- and 96-week-old mice, respectively, versus 7.08% in 8-week-old mice) and in the thoracic aorta (-1.89%, -5.23% and -14.03% for 36-, 48- and 96-week-old mice, respectively, versus 7.81% in 8-week-old mice). The impairment of endothelial function in the FA in vivo, assessed as flow-mediated vasodilation, was also observed in 36-week-old mice, but further changes in older mice progressed less with age in comparison to impairment of endothelium-dependent vasodilation observed in the aorta (Fig. 1E). Importantly, the endothelium-independent response induced by SNP in the AA and TA was unchanged during ageing in C57BL/6 mice (Fig. 1C), confirming the occurrence of specific, age-dependent deterioration of endothelium-dependent vasodilation with fully preserved endothelium-independent vasodilator function.

Age-dependent progression of endothelial dysfunction was associated with increased arterial stiffness, measured as PWV in the AA and TA (Fig. 1D). However, PWV significantly increased only in the oldest mice, at the age of 48- and 96-weeks, suggesting the arterial stiffness may be a delayed phenotypic parameter of aged vasculature as compared with age-dependent endothelial dysfunction.

### Epigenetic age prediction in blood and in aortic samples

**Fig. 2:**
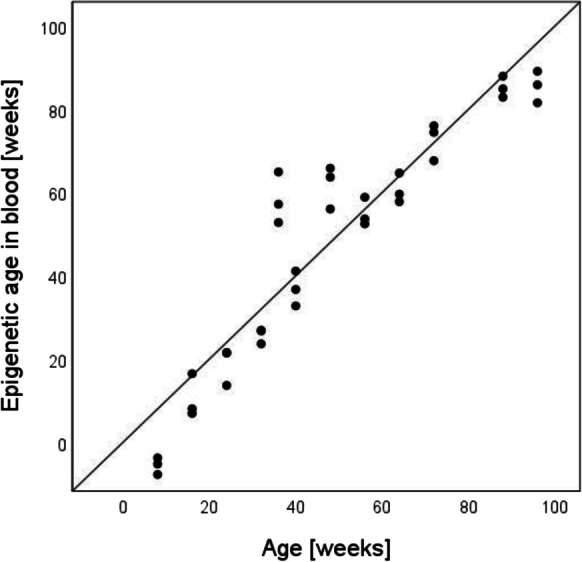
Age prediction in mouse blood samples (*N* = 36) using three cytosines and a clock by Han et al. [19]**.** The literature model predicted age based on C57BL/6 blood samples with MAE = 7.9 weeks.

**Fig. 3:**
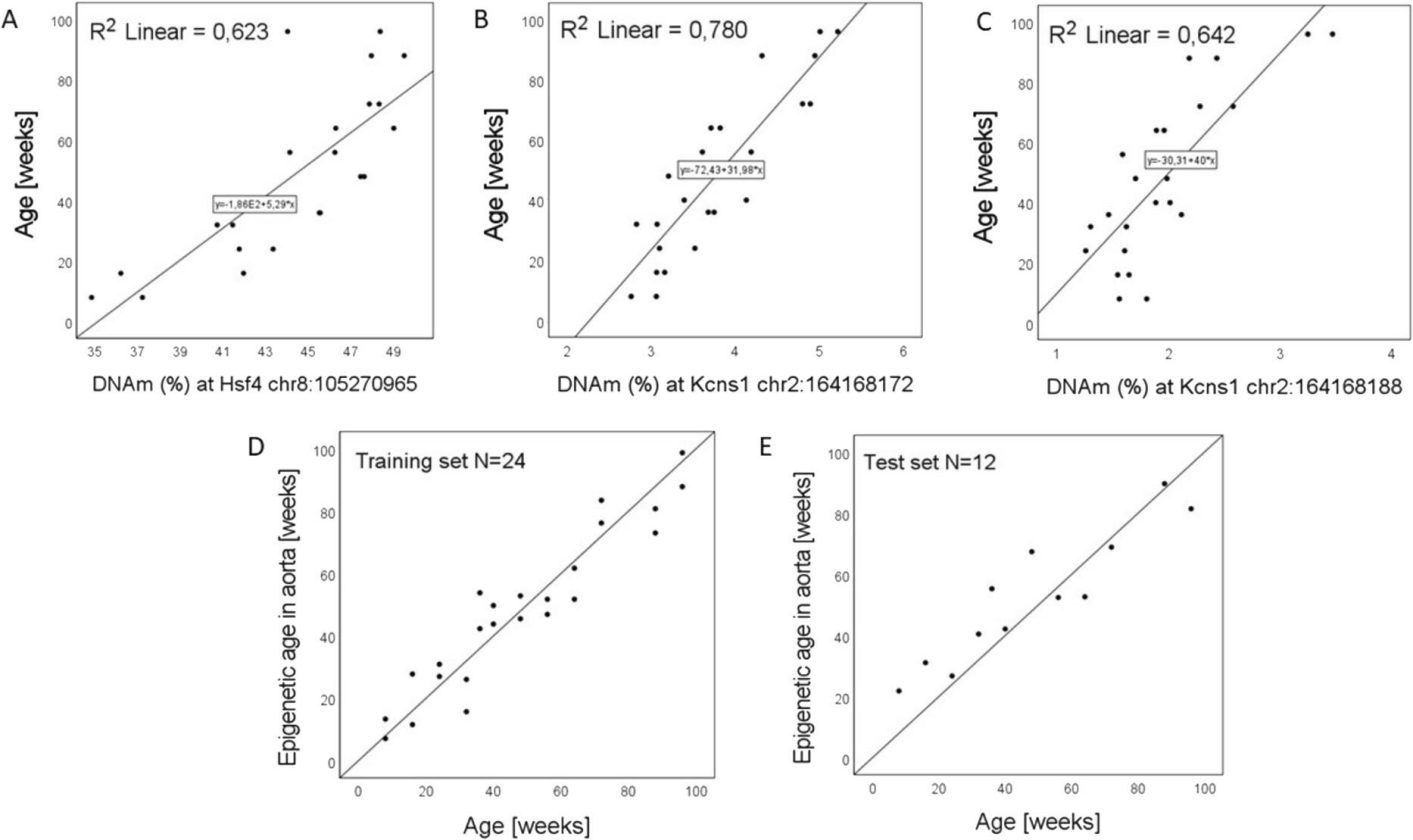
Novel epigenetic biomarker of aortic age. The graph shows the correlation between age and DNA methylation (DNAm) at three CpG sites selected for modelling (**A**—**C**) and predicted vs. chronological age in the training (**D**) and test (**E**) sets. The developed model predicted age in C57BL/6 aortic samples, with MAE = 7.3 and 9.6 weeks, for training and test sets, respectively.

DNA methylation at 41 CpGs across three loci (*Prima1*, *Kcns1* and *Hsf4*) was measured in aorta and blood samples from 36 C57BL/6 mice and was analyzed in three biological replicates at 12 time points in mice between 8 and 96 weeks of age using an amplicon-based enrichment protocol and high-throughput sequencing. The DNAm data for blood (*N* = 36) were used to validate the three-cytosine model from Han et al. (*Prima1* chr12:103,214,640, *Hsf4* chr8:105,271,022, *Kcns1* chr2:164,168,063) and trained on bisulfite amplicon sequencing data as described in [21]. A high correlation of the prediction results with chronological age was observed (r = 0.94, *p* = 6.8 × 10^–17^), and the accuracy of the prediction, as described by an MAE, was 7.9 weeks (Fig. 2). However, a much weaker correlation (r = 0.38, *p* = 0.02) of prediction results with chronological age was observed for aortic samples when using the original clock by *Han* et al. [21]. Therefore, we used the data for all 41 CpGs to develop a new model for age estimation based on aorta samples by regressing the best performing cytosines with chronological age in *N* = 24 aortic samples included in the training set. Multivariate linear regression analysis led to the selection of three cytosines in *Kcns1* and *Hsf4* ( *Hsf4* chr8:8,105,270,965 *p* = 4.5 × 10^–4^, *Kcns1* chr2:2,164,168,172 *p* = 0.03, *Kcns1* chr2:2,164,168,188 *p* = 0.02), which together explained R^2^ = 87.8% of the observed variation in age in the aortic samples analysed (Fig. 3A-C). The developed biomarker was described using following regression equation: Epigenetic age in aorta =—153.142 + 2.744* *Hsf4* chr:8,105,270,965 + 12.589* *Kcns1* chr:2,164,168,172 + 16.463* *Kcns1* chr:2,164,168,188. It predicted age of C57BL/6 mice with an accuracy described by MAE of 7.3 weeks in the training set (*N* = 24) and 9.6 weeks in the independent test set (*N* = 12) (Fig. 3D-E).

### Association of epigenetic age acceleration with endothelial function

For both, the Han et al. model for blood [21] and the novel estimator of aortic age, the epigenetic age acceleration parameter was calculated and a positive association of EAA in blood (*p* = 0.002) and in the aorta (*p* = 0.03) with impaired endothelial function in the AA was observed. Furthermore, EAA was also associated with impaired FA function (*p* = 0.003 and *p* = 0.02, for blood and aorta, respectively). Interestingly, this effect was not significant for TA and was not consistent when aortic stiffness was used as a readout (Table 1).

**Table 1:** Association analysis between epigenetic age acceleration (EAA) and endothelial dysfunction traits in C57BL/6 mice. Results are adjusted for chronological age. EAA was measured using the Han et al. clock [19] for blood samples and a novel biomarker of age for aortic samples. SE - Standard Error; Stand. beta - Standardized beta coefficient

Tissue	EAA measurement method	beta	SE	Stand. beta	t	*p*-value
Endothelial dysfunction in the abdominal aorta (AA)						
Blood *N* = 15	Han et al. clock	-0.201	0.051	-0.308	-3.936	**0.002**
Aorta *N* = 15	Novel biomarker	-0.285	0.112	-0.242	-2.541	**0.026**
Endothelial dysfunction in the thoracic aorta (TA)						
Blood *N* = 15	Han et al. clock	-0.113	0.052	-0.188	-2.180	0.050
Aorta *N* = 15	Novel biomarker	-0.159	0.099	-0.148	-1.602	0.135
Endothelial dysfunction in the femoral artery (FA)						
Blood *N* = 15	Han et al. clock	-0.205	0.055	-0.436	-3.721	**0.003**
Aorta *N* = 15	Novel biomarker	-0.313	0.114	-0.371	-2.754	**0.017**
Stiffness of the abdominal aorta						
Blood *N* = 15	Han et al. clock	-0.003	0.007	-0.061	-0.447	0.663
Aorta *N* = 15	Novel biomarker	0.001	0.013	0.009	0.065	0.950
Stiffness of the thoracic aorta						
Blood *N* = 15	Han et al. clock	-0.015	0.004	-0.297	-3.959	**0.002**
Aorta *N* = 15	Novel biomarker	-0.017	0.009	-0.190	-1.903	0.081

## Discussion

Age-dependent decline of endothelial function predicts CVD-related morbidity and mortality [6, 7], but the mechanisms responsible for the ageing of vasculature are far from well-understood [6]. Notably, despite substantially similar burdens of cardiovascular risk factors, vascular age in humans may differ from chronological age, as evidenced by the presence of SUPERNOVA or EVA (Early and Supernormal Vascular Ageing) phenotypes of vascular ageing [25]. Therefore, there is a need to define factors that could contribute to accelerated vascular ageing. Here, we verified epigenetic ageing based on the analysis of selected DNA methylation markers, and tested whether epigenetic clock could be a useful tool for predicting age-dependent endothelial dysfunction.

To assess endothelial function, we collected 3D MRI-based measurements in vivo. This methodology has been shown to be well suited for quantification, with good sensitivity and reproducibility of endothelial function [22, 23, 26]. Notably, this in vivo MRI-based method of assessing endothelial function has been found to be more sensitive for detecting endothelial dysfunction as compared to analysis of endothelium-dependent vasodilation ex vivo in myograph [26] or measurement of NO production in the aorta [22].

Using this approach, we demonstrated age-dependent development of endothelial dysfunction in C57BL/6 mice an age of 36 weeks or older. Next, we successfully validated the original clock method by Han et al. [21] and developed a new 3-CpG model for assessing age with aorta sampling using a modified set of CpGs located in *Kcns1* and *Hsf4*, which were selected as the best performing CpGs through multivariate regression analysis. All three cytosines included in the biomarker of aortic age showed a strong correlation with chronological age (r = 0.79—0.88, *p* = 2.56 × 10^–6^—1.1 × 10^–8^ in univariate association tests), although their observed methylation ranges were relatively narrow (Fig. 3A-C). Moreover, through analysis of EAA association we demonstrated that both models were sensitive to age-dependent changes in endothelial function in the aorta. Interestingly, the epigenetic clock in the aorta correlated with age-dependent endothelial dysfunction in the AA and FA, but was not significant in the TA, which may suggest that ageing in the TA can be mechanistically different as compared with the AA. This notion seems to be compatible with a different perivascular adipose tissue depot in the TA and AA [23]. Although arterial stiffness is an important phenotypic feature of ageing vasculature, in the present study it appeared to be delayed in comparison with age-dependent endothelial dysfunction, much like in our previous work [23], this might explain the lack of the correlation between our epigenetic clock in the aorta and arterial stiffness’ parameter (Pulse Wave Velocity).

It needs to be underlined, that in our study, the rate of vascular biological ageing in mice was estimated using DNA methylation measurements only during normal ageing process. Therefore, further experiments are needed, in which monitoring of vascular biological ageing will be performed in pathological conditions or in mice after interventions, in particular as EAA measurements have becoming increasingly important in clinical trials [27]. The study is also limited by the relatively small number of samples per group, therefore future independent validation is recommended. However, the selection of markers based on literature data and the high correlation and statistical significance of individual CpG markers with chronological age support the reliability of the results presented in this report.

In summary, the murine epigenetic clock for the aorta developed here as well as in blood samples using DNA methylation measurements at three CpG sites in the *Prima1*, *Hsf4* and *Kcns1* genes reflect the age-dependent deterioration of systemic endothelial function and thus rate of vascular biological ageing and provides a useful tool for monitoring vascular biological ageing in mice. In particular, given that various dietary or pharmacological treatments can regulate the rate of epigenetic ageing and susceptibility to age-related diseases [17, 20, 28], this novel tool may be useful for studies aimed at rejuvenating ageing vasculature in mice. Further studies are however warranted to verify whether, this novel tool will provide a useful and a reliable parameter to monitor rejuvenating effects of experimental interventions on epigenetic ageing and age-dependent deterioration of endothelial function in mice.

## Data Availability

The data, analytic methods, and study materials will be made available on request to other researchers for purposes of reproducing the results or replicating the procedure.

## Abbreviations

**AA**: Abdominal aorta
**Ach**: Acetylcholine
**CVD**: Cardiovascular disease
**DNAm**: DNA methylation
**EAA**: Epigenetic age acceleration
**FA**: Femoral artery
**FMD**: Flow-mediated dilatation
**MAE**: Mean absolute error
**MRI**: Magnetic resonance imaging
**NO**: Nitric oxide
**SNP**: Sodium nitroprusside
**PWV**: Pulse wave velocity
**TA**: Thoracic aorta
